# Evaluation of the Awareness, Beliefs, and Psychological Impact of Patients with Alopecia Areata in Makkah City, Saudi Arabia

**DOI:** 10.1155/2023/4286891

**Published:** 2023-05-23

**Authors:** Basant A. Alzubaidy, Tasneem A. Banjar, Murouj A. Almaghrabi, Shahad S. Alkidaiwi, Lena M. Basfar, Khlood A. Alzubaidy, Safaa K. Dhafar, Aymen Alharbi

**Affiliations:** ^1^College of Medicine, Umm Al-Qura University, Makkah, Saudi Arabia; ^2^Ministry of Health Holdings, Makkah, Saudi Arabia; ^3^College of Medicine, Ibn Sina National College, Jeddah, Saudi Arabia; ^4^Family Medicine Resident Makkah Joint Program, Makkah, Saudi Arabia; ^5^Consultant Dermatologist and Hair Disorders and Hair Transplantation, Chairman of the Local Committee of Dermatology Training in the Western Region, Makkah, Saudi Arabia

## Abstract

**Methods:**

A descriptive cross-sectional study was conducted among patients diagnosed with AA at different hospitals. A total of 211 patients were included in the investigation between November 2021 and June 2022. The participants were subjected to a well-structured, Arabic-based, validated questionnaire.

**Results:**

More than half of the patients were men (54.0%) and single (50.7%). The most common age groups were 30 to 44 years (46.9%) and 18 to 29 years (36.0%). More than two-thirds of the participants (67.8%) had heard of AA before their diagnosis. Of these, the level of knowledge was low among 36.4%, medium among 42.0%, and high among 21.7%. Most of the patients believed that AA may be caused by genetic, nutritional, or health factors (77.3%) and that it is a serious health problem that necessitates treatment (64.0%). The most common psychological attributes caused by AA are feeling ashamed in front of other individuals (63.0%), anxiety (47.9%), and depression (36.0%).

**Conclusion:**

Our results show more than two-thirds of the participants were knowledgeable about AA. Most believed that the cause of AA is genetic, nutritional, or health factors and suffered negative psychological effects. According to our study, high levels of anxiety and depression are closely associated with AA patients, which affect their quality of life.

## 1. Introduction

Alopecia areata (AA) is an autoimmune hair disorder [1]. It is characterized by round or oval noncicatricial patches of hair loss on the scalp or the body that may be revisable [2]. Its severity runs from small patches on the scalp to complete scalp hair loss (alopecia totalis) or the entire loss of scalp and body hair (alopecia universalis). AA is one of the most prevalent hair loss types, with a global incidence of approximately 2% [3]. In Saudi Arabia, the prevalence of the disease is higher than in western countries which may be due to the high consanguinity rate in Saudi Arabia [4].

AA demands a clinical-based diagnosis; however, a skin biopsy can be used in certain situations [5]. Family history and genetic factors play a major role in the pathogenesis of the disease and the environmental insult or psychosocial stress that affect its progression. As a lifelong disease, it has a significant effect on patients' emotional and psychological well-being. Patients with AA may experience anxiety, depression, and other psychiatric stress, which decreases their quality of life [6]. While AA is incurable, different modalities might help improve the condition, including topical treatment, local injections, systemic steroid and topical immunotherapy, minoxidil or anthralin, systemic immunosuppressants such as cyclosporine or methotrexate [3], and Janus kinase inhibitors such as tofacitinib and baricitinib.

Despite substantial progress in understanding the etiology of AA and its treatment options, the lack of knowledge and poor behaviors of patients with AA are roadblocks to successful treatment. Several studies have reported remarkably low levels of knowledge, beliefs, and perceptions of patients with AA, including studies in Pakistan [7], the Philippines [8], the United States [9], and Qassim, Saudi Arabia [10]. Coping with this hair loss experience is influenced by the patient's understanding, perception, and knowledge of AA, which leads to greater adjustment. Ignorance and illogical conduct, on the other hand, amplify the harmful effects of AA [11].

Because available data on the awareness, knowledge, and perception of AA patients are limited in western Saudi Arabia, the current study aims to evaluate the awareness, beliefs, and psychological impact of patients with AA in Makkah, Saudi Arabia.

## 2. Materials and Methods

### 2.1. Study Design and Population

A descriptive cross-sectional web-based study was conducted between November 2021 and June 2022 using an online questionnaire which designed in Google forms and distributed among patients with AA in Makkah. The authors reached those patients by reviewing the medical records in the dermatology department of three random hospitals in Makkah. We asked both genders above 18 years with a confirmed diagnosis of AA to complete the questionnaire. We excluded patients from any medical/health specialties (including medicine and surgery, dentistry, physiotherapy, laboratory medicine, and clinical nutrition) and those who refused to consent to participate in the study.

The minimum sample size required for this study was calculated by OpenEpi version 3.0 [12], which uses the following equation: *n* = [DEFF *∗* Np(1 − *p*)]/[(*d*^2^/*Z*^2^_1 − *α*/2_ *∗* (*N* − 1) + *p* *∗* (1 − *p*)], and we considered the population size of patients with AA as 741 patients based on the prevalence of patients with AA in Saudi Arabia [4]. Keeping the confidence interval (CI) at 95% and the design effect at 1.0, and considering the anticipated frequency as 10% [10], the sample size was calculated to be 117 participants. Nevertheless, the final number of included participants reached 211 patients.

### 2.2. Study Tool

Data from patients with AA were recorded using an online questionnaire. The participants were subjected to a well-structured, Arabic-based, validated questionnaire. The questionnaire was inspired by a previous study conducted among 50 patients and published in July 2021 [7]. The questionnaire consists of five sections. Section one included patients' consent and agreement to participate in the study. Section two asked about demographic information, including age, gender, educational level, and marital status. Section three concentrated on the assessment of AA awareness. Section four asked patients to state their beliefs about AA. The last section was concerned with the factors and psychological impacts of AA.

### 2.3. Validity and Reliability of the Questionnaire

The questionnaire was first validated using content validity, with an assessment conducted by content experts. Thereafter, face validity was assessed by a medical educationist who found that the survey met the objectives of the survey and that the flow of the questions was in a logical sequence. To test the reliability of the questionnaire, a pilot study was conducted with 40 participants; the questionnaire was then modified according to the respondents' feedback. Cronbach's alpha was calculated as above 0.7, which suffices for reliability.

### 2.4. Statistical Analysis

Statistical analysis was conducted using RStudio (R version 4.1.1). Frequencies and percentages were used to express the categorical variables. Items with multiple responses were analyzed using the multiple response analysis technique. To determine the level of knowledge, multiple choice questions were used, and the right answers were given a score of “1,” while the wrong answer or “I don't know” answers were given a score of “0.” Participants who correctly answered 10–20% of the questions were labeled as “low level,” 30–50% as “medium level,” and >50% as “high level.” Factors associated with knowledge on AA were assessed using Pearson's Chi-squared test or Fisher's exact test whenever applicable. The significantly associated factors were further used as independent variables in a binary logistic regression model to investigate the independent predictors of favorable AA knowledge. The results of the regression analysis were presented as the odds ratio (OR) and respective 95% CI. A *p* value of <0.05 indicated statistical significance.

### 2.5. Ethical Considerations

The Institutional Review Board (IRB) at King Abdulaziz Hospital, Makkah, Saudi Arabia, approved this study (IRB No. H-02-K-076-1021-584 Date: 2.11.2021). All the participants were provided with information on the study before answering the questionnaire. The collected data were stored securely for research purposes, and electronic informed consent was obtained once the patient was approved to complete the questionnaire.

## 3. Results

### 3.1. Sociodemographic Characteristics

We received responses from 211 patients with AA. More than half of the patients were men (54.0%) and single (50.7%). The most common age categories were 30 to 44 years (46.9%) and 18 to 29 years (36.0%). Patients with a bachelor's degree or diploma represented 58.8% of the sample (Table 1).

### 3.2. Source of Information on AA

**Figure 1:**
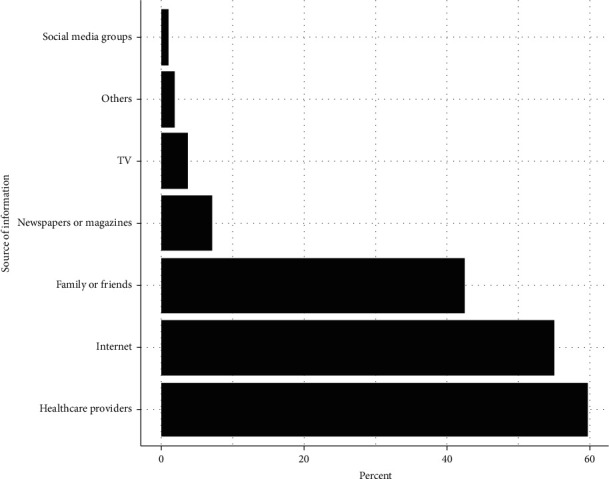
The percentage of participants' responses regarding the sources of information about alopecia areata.

The participants indicated that the most common sources of information were healthcare providers (59.7%) and the Internet (55.0%). On the other hand, the least common sources of information were social media groups (0.9%) and other sources (1.9%), which included information retrieved from other experienced individuals, scientific research, and clinics (Figure 1).

### 3.3. Characteristics of Disease Diagnosis and Management Approaches

The majority of the respondents revealed that they had been diagnosed with AA at dermatology clinics (67.3%), whereas approximately one-third were self-diagnosed (35.1%). About half of the participants (50.2%) were directly referred to a dermatology clinic by a hospital, and the remainder were referred by a health center. More than three-quarters of the patients with AA (76.3%) had sought to visit hospitals and healthcare centers for the management of AA. By comparison, home remedies and traditional medicine were used by 59.7% (Table 2).

### 3.4. Knowledge on AA and Associated Factors

More than two-thirds of the participants (67.8%) had heard of AA before their diagnosis. Of these, the level of knowledge was low (10–20%) among 36.4% of the respondents, medium (30–50%) among 42.0%, and high (more than 50%) among 21.7%. Notably, 27.5% of the respondents stated that they had a family member diagnosed with AA.

Regarding the factors associated with the respondents' knowledge on AA before their diagnosis, the results showed that knowledge was associated with educational level, with significantly higher proportions of uneducated participants (87.5%) and those who had obtained a bachelor's degree or diploma (73.4%) having knowledge on AA than those at other educational levels (*p*=0.028). Furthermore, a significantly higher proportion of married participants had AA knowledge than those in other relationship categories (*p*  <  0.001, Table 3). However, the results of the multivariate logistic regression analysis indicated that being married was the only significant predictor of favorable knowledge on AA (OR = 3.05, 95% CI = 1.59 to 6.03, *p*  <  0.001, Table 4).

### 3.5. Beliefs about AA and Its Impact on Quality of Life

The majority of the patients believed that AA may be caused by genetic, nutritional, or health factors (77.3%) and that it is a serious health problem that necessitates treatment (64.0%). Additionally, 75.4% of the respondents thought that an effective treatment for the disease existed. Concerning the influence of AA on the participants' lives, only 37.4% thought that it is difficult to live with AA and 40.8% indicated that the disease might alter their quality of life (Table 5).

### 3.6. Psychological Effects of AA

A considerable proportion of the participants declared that AA negatively affects the psychological side (79.6%). The most common psychological attributes caused by AA were feeling ashamed in front of other individuals (63.0%), anxiety (47.9%), and depression (36.0%, Table 6).

## 4. Discussion

Available data on the awareness, knowledge, and perception of AA patients are limited in Western of Saudi Arabia. Hence, The Kingdom of Saudi Arabia is characterized by the large geographical area and the different traditions in each region. As instant, the consanguineous marriage is known to be higher in some regions compared to others, which affects the rates of disease, respectively. Accordingly, our current study focused on studying one of the vast regions of the Kingdom. This descriptive cross-sectional study aimed to evaluate the level of awareness, beliefs, and psychological impact of patients with AA in Makkah, Saudi Arabia. The findings showed that the level of knowledge was low among 36.4% of the respondents, medium among 42.0%, and high among 21.7%. Furthermore, the largest proportion of the participants believed that AA may be caused by genetic, nutritional, or health factors and that it is a serious health problem that may affect quality of life (40.8%); only 37.4% of them thought that it is difficult to live with AA. A large proportion of the participants declared that AA negatively affects the psychological side and stated that the most common psychological attributes caused by AA are feeling ashamed in front of other individuals, followed by anxiety and depression.

In our study, we reported that AA is slightly more prevalent among men than women; previous studies have reported similar findings [4, 7, 9]. However, the latest article published by the National Institute of Arthritis and Musculoskeletal and Skin diseases reported that both genders might develop AA with an equal chance [13]. Regarding patients' sources of information on AA, our study indicated that the most common sources were healthcare providers (59.7%) followed by the Internet (55.0%). These findings demonstrate the huge dependence on the Internet. Several studies have found that the rising amount of OHI may affect the physician–patient relationship by influencing patient behaviors [14, 15]. Although OHI does make patients aware and knowledgeable of their condition, enhance their decision-making, and improve their self-confidence when communicating, an uneven quality of OHI may lower patients' understanding and health behaviors [16]. The key factor to ensuring that OHI benefits the physician–patient relationship is to enhance people's health information literacy and the quality of OHI.

Regarding demographics, marital status was a significant factor, as married participants had higher levels of knowledge (*p*  <  0.001). Although the association between marital status or age and the risk of AA is unclear, it has been observed that elderly patients, especially women, are more susceptible to developing AA after sustaining severe trauma or bleeding or suffering psychological stress [17]. By contrast, studies have reported the incidence in children to be around 2.7% [18]. Higher levels of knowledge among married participants may be age related or due to the higher levels of AA among their social circles, which makes them more vulnerable to gain knowledge about the disease.

A previous study found that the impact of AA on quality of life was more profound within the married category than among the unmarried. These results showed that the greater number of responsibilities among the married make them worry, leading to psychological distress [7]. Our study showed that 37.4% of the participants thought that it is difficult to live with AA, while 40.8% indicated that the disease may alter their quality of life (Table 5). In addition, the majority of our participants suffer lower psychological health as a result (79.6%). Feeling ashamed in front of people is the major psychological effect in our study (63.0%), followed by anxiety (47.9%).

The dilemma of the impact of AA on quality of life has been widely studied globally. Previous meta-analyses have been carried out in the United States, Italy, France, Brazil, Belgium, China, Korea, Japan, Turkey, Iran, Tunisia, and Kuwait. These studies have revealed that AA patients have significantly impaired health-related quality of life, especially mental health [19]. The disease not only has a severe psychological impact but also causes a marked disturbance in the social life of patients, forcing them to avoid social gatherings, change their hairstyle, and alter the type of clothing they wear. Compared with the general population, AA patients presented a significantly altered quality of life, where patients with a 51% to 75% hair loss showed significantly lower scores on the social functioning subscale than those with a hair loss of less than 25% [11]. Thus, paying attention to the psychological sensitivity of AA patients is crucial. In addition, offering a suitable psychiatric/psychological intervention, including psychotherapy, relaxation techniques, antidepressants, group therapy, and hypnosis, might be a life changer [20–22].

Our study reported that more than two-thirds of the participants (67.8%) had heard of AA before their diagnosis. Of these, 42.0% rated their level of knowledge as medium (30–50%). Muhammad and colleagues reported that the majority (62%) had no knowledge on what causes AA (*p*  <  0.05) and that 30% considered it to be a serious health issue. A significantly (*p*  <  0.05) higher number of patients (78%) perceived that AA has no association with diet and almost half (42%) of participants believed that germs and viruses cause AA [7]. Al-ajlan et al. noted that family history is an important factor in AA, as 37% of their participants with AA also had a first-degree relative with the condition [4]. Our study supports the finding that family history is an important factor in AA. Notably, 27.5% of our respondents stated that they had a family member diagnosed with AA.

Our study represents an evaluation of the awareness, beliefs, and psychological impact of patients with AA in Makkah, which demonstrated a good level of knowledge but high levels of anxiety and depression. This finding has a great clinical implication for the crucial need for psychiatric/psychology integration in the management of AA patients which may, in turn, reflect on their quality of life. Our study includes several limitations to disclose. First, an online platform-based study may limit the generalizability of the study's results. Second, reporting bias is possible as our study depends on self-reported information of the patients. Lastly, the current study used a cross-sectional design which precludes the ability to deeply understand the causation between associated variables.

## 5. Conclusion

Our results show that more than two-thirds of the participants were knowledgeable about AA. Most believed that the cause of AA was genetic, nutritional, or health factors and suffered negative psychological effects. According to our study, high levels of anxiety and depression are closely associated with AA patients which affect their quality of life. As AA is one of the most common hair disorders, adequate psychological evaluation for patients with AA is needed to determine the effects of the disease. This article can be a preliminary step toward understanding the psychological impact, beliefs, and awareness of AA patients.

## Figures and Tables

**Table 1:** Sociodemographic characteristics.

Parameters	Category	*N* (%)
Gender	Male	114 (54.0%)
	Female	97 (46.0%)

Age	<18	24 (11.4%)
	18 to <30	76 (36.0%)
	30 to <45	99 (46.9%)
	45 to <60	9 (4.3%)
	60 or more	3 (1.4%)

Educational level	Uneducated	8 (3.8%)
	High school or less	71 (33.6%)
	Bachelor or diploma	124 (58.8%)
	Postgraduate	8 (3.8%)

Social status	Single	107 (50.7%)
	Married	96 (45.5%)
	Divorced	7 (3.3%)
	Widow	1 (0.5%)

**Table 2:** Characteristics of disease diagnosis and the management approaches.

Parameters	Category	*N* (%)
How have you been diagnosed with alopecia areata?^*∗*^	Self-diagnosed	74 (35.1%)
	Dermatology clinic	142 (67.3%)
	Health center	50 (23.7%)
	Family members	37 (17.5%)

How did you get referred to the dermatology clinic?	Directly via a hospital	106 (50.2%)
	Referred from a health center	105 (49.8%)

What are the methods which you used to treat alopecia areata?^*∗*^	Going to the hospital/health center	161 (76.3%)
	Consulting a pharmacist	39 (18.5%)
	Home remedies and traditional medicine	126 (59.7%)
	Get your hair cut	34 (16.1%)
	Others	5 (2.4%)

^*∗*^An asterisk indicates a multiple response item.

**Table 3:** Factors associated with participants' knowledge regarding alopecia areata before diagnosis.

Parameters	Category	Knowledge about AA		
		No, *N* = 68	Yes, *N* = 143	*p*
Gender	Male	40 (35.1%)	74 (64.9%)	0.335
	Female	28 (28.9%)	69 (71.1%)	

Age	<18	8 (33.3%)	16 (66.7%)	0.226
	18 to <30	31 (40.8%)	45 (59.2%)	
	30 to <45	27 (27.3%)	72 (72.7%)	
	45 to <60	1 (11.1%)	8 (88.9%)	
	60 or more	1 (33.3%)	2 (66.7%)	

Educational level	Uneducated	1 (12.5%)	7 (87.5%)	**0.028**
	High school or less	29 (40.8%)	42 (59.2%)	
	Bachelor or diploma	33 (26.6%)	91 (73.4%)	
	Postgraduate	5 (62.5%)	3 (37.5%)	

Social status	Single	46 (43.0%)	61 (57.0%)	**<0.001**
	Married	19 (19.8%)	77 (80.2%)	
	Divorced	2 (28.6%)	5 (71.4%)	
	Widow	1 (100.0%)	0 (0.0%)	

Bold values represent statistical significance data as *p* value of  < 0.05.

**Table 4:** Results of the regression analysis to assess the predictors of AA knowledge before diagnosis.

Parameters	Category	OR	95% CI	*p*
Social status	Single	Ref	Ref	
	Married	3.05	1.59, 6.03	**<0.001**
	Divorced	1.53	0.30, 11.2	0.628
	Widow	NA	NA	0.990

Educational level	High school or less	Ref	Ref	
	Uneducated	NA	NA	0.985
	Bachelor or diploma	1.59	0.83, 3.06	0.161
	Postgraduate	0.28	0.05, 1.31	0.114

NA: the record was nonavailable because one arm had a zero or one value. Bold values represent statistical significance data as *p* value of  < 0.05.

**Table 5:** Beliefs about alopecia areata and its impact on the quality of life.

Parameters	Category	*N* (%)
Alopecia areata is a serious health problem that needs to be treated	Yes	135 (64.0%)

There are genetic, nutritional, or health factors that may cause alopecia areata	Yes	163 (77.3%)

There is an effective treatment for alopecia areata	Yes	159 (75.4%)

It is difficult to live with alopecia areata	No	47 (22.3%)
	May be	85 (40.3%)
	Yes	79 (37.4%)

Alopecia areata may alter the quality of daily life	No	52 (24.6%)
	May be	73 (34.6%)
	Yes	86 (40.8%)

**Table 6:** Psychological effects of alopecia areata.

Parameters	Category	*N* (%)
Alopecia areata affects the psychological side in a negative way	Yes	168 (79.6%)

Negative psychological effects associated with alopecia areata^*∗*^	Anxiety	101 (47.9%)
	Feeling ashamed in front of people	133 (63.0%)
	Depression	76 (36.0%)
	Nervousness	60 (28.4%)
	I am fine and I do not feel any thing	41 (19.4%)
	Other	9 (4.3%)

## Data Availability

The data supporting the current study are available from the corresponding author upon request.
